# Triaxial bearing vibration dataset of induction motor under varying load conditions

**DOI:** 10.1016/j.dib.2022.108315

**Published:** 2022-05-23

**Authors:** Dileep Kumar, Sanaullah Mehran, Muhammad Zakir Shaikh, Majid Hussain, Bhawani Shankar Chowdhry, Tanweer Hussain

**Affiliations:** NCRA Condition Monitoring Systems Lab, Mehran University of Engineering and Technology, Jamshoro, Pakistan

**Keywords:** Bearing, Triaxial Vibration, Condition Monitoring, Induction motor, Fault diagnosis

## Abstract

Rotating machines as core component of an industry can effectively be monitored through vibration analysis. Considering the importance of vibration in industrial condition monitoring, this article reports and discusses triaxial vibration data for motor bearing faults detection and identification. The data is acquired using a MEMS based triaxial accelerometer and the National Instruments myRIO board. The bearing conditions include healthy bearing, bearings with inner race faults, and bearings with outer race faults. For each faulty bearing condition, the three-phase induction motor is operated under three different load conditions. The dataset can be used to assess performance of newly developed methods for effective bearing fault diagnosis. Mendeley Data. http://dx.doi.org/10.17632/fm6xzxnf36.2.

## Specifications Table

**Table:** 

Subject	Mechanical Engineering
Specific subject area	Motor Condition Monitoring
Type of data	Time-series triaxial vibration data Tables Figures
How the data were acquired	The triaxial vibration data was acquired through a MEMs based accelerometer and myRIO board.
Data format	Raw
Description of data collection	The data was acquired under 12 different bearing conditions. Each bearing condition was experimented with the three-phase induction motor operated under the three different load conditions including 100W, 200W, and 300W loads.
Data source location	Institution: NCRA Condition Monitoring Systems Lab, Mehran University of Engineering and Technology City: Jamshoro Country: Pakistan
Data accessibility	Repository name: Triaxial Bearing Vibration Dataset of Induction Motor under Varying Load Conditions Data identification number (doi): 10.17632/fm6xzxnf36.2 Direct link to the dataset: http://dx.doi.org/10.17632/fm6xzxnf36.2

## Value of the Data

• The dataset is acquired under the varying load conditions and it is different from available datasets. Moreover, it is a triaxial vibration dataset which represents the impact of bearing faults on the axial vibrations of the motor.
• The dataset can be used to analyse the time and frequency characteristics of bearings under varying load conditions.
• This dataset can be used to benchmark newly developed methods such as Machine Learning/Deep Learning algorithms implemented for motor condition monitoring or bearing fault diagnosis.

## 1 Data Description

The collected data includes triaxial vibration data of bearings operated under different load conditions. It also includes triaxial vibration data of three phase induction motor in healthy condition. The acquired data can be used for evaluation of new methods proposed for bearing fault detection and identification such as methods presented in the research [1], [2], [3], [4]. The collected datasets are stored in comma separated values (CSV) files. Each file contains four columns namely ‘Time Stamp’, ‘X-axis’, ‘Y-axis’, and ‘Z-axis’. The first column includes the time of acquired data samples in seconds. The second, third, and fourth column contains vibration data of the motor along the x, y, and z axes. The unit of the vibration along the three axes is ‘g’ (1g = 9.80665 m/s^2^). Each file contains different data acquired under various motor operating and health conditions. The description of the files as per operating and health conditions of the motor are provided as follows:

1. Healthy-without-pulley.csv: This file includes trixial vibration data of the bearing in healthy condition acquired from the motor without connected to load.
2. Healthy-with-pulley.csv: This file includes trixial vibration data of the bearing in healthy condition acquired from the motor with connected to load.
3. 0.7inner-100 watt: Triaxial vibration data of the bearing with inner race fault of 0.7 mm and the motor operated under the load of 100watt.
4. 0.7inner-200 watt: Triaxial vibration data of the bearing with inner race fault of 0.7 mm and operated the motor under the load of 200watt.
5. 0.7inner-300 watt: Triaxial vibration data of the bearing with inner race fault of 0.7 mm and the motor operated under the load of 300watt.
6. 0.7outer-100 watt: Triaxial vibration data of the bearing with outer race fault of 0.7 mm and the motor operated under the load of 100watt.
7. 0.7outer-200 watt: Triaxial vibration data of the bearing with outer race fault of 0.7 mm and the motor operated under the load of 200watt.
8. 0.7outer-300 watt: Triaxial vibration data of the bearing with outer race fault of 0.7 mm and the motor operated under the load of 300watt.
9. 0.9inner-100 watt: Triaxial vibration data of the bearing with inner race fault of 0.9 mm and the motor operated under the load of 100watt.
10. 0.9inner-200 watt: Triaxial vibration data of the bearing with inner race fault of 0.9 mm and the motor operated under the load of 200watt.
11. 0.9inner-300 watt: Triaxial vibration data of the bearing with inner race fault of 0.9 mm and the motor operated under the load of 300watt.
12. 0.9outer-100 watt: Triaxial vibration data of the bearing with outer race fault of 0.9 mm and the motor operated under the load of 100watt.
13. 0.9outer-200 watt: Triaxial vibration data of the bearing with outer race fault of 0.9 mm and the motor operated under the load of 200watt.
14. 0.9outer-300 watt: Triaxial vibration data of the bearing with outer race fault of 0.9 mm and the motor operated under the load of 300watt.
15. 1.1inner-100 watt: Triaxial vibration data of the bearing with inner race fault of 1.1 mm and the motor operated under the load of 100watt.
16. 1.1inner-200 watt: Triaxial vibration data of the bearing with inner race fault of 1.1 mm and the motor operated under the load of 200watt.
17. 1.1inner-300 watt: Triaxial vibration data of the bearing with inner race fault of 1.1 mm and the motor operated under the load of 300watt.
18. 1.1outer-100 watt: Triaxial vibration data of the bearing with outer race fault of 1.1 mm and the motor operated under the load of 100watt.
19. 1.1outer-200 watt: Triaxial vibration data of the bearing with outer race fault of 1.1 mm and the motor operated under the load of 200watt.
20. 1.1outer-300 watt: Triaxial vibration data of the bearing with outer race fault of 1.1 mm and the motor operated under the load of 300watt.
21. 1.3inner-100 watt: Triaxial vibration data of the bearing with inner race fault of 1.3 mm and the motor operated under the load of 100watt.
22. 1.3inner-200 watt: Triaxial vibration data of the bearing with inner race fault of 1.3 mm and the motor operated under the load of 200watt.
23. 1.3inner-300 watt: Triaxial vibration data of the bearing with inner race fault of 1.3 mm and the motor operated under the load of 300watt.
24. 1.3outer-100 watt: Triaxial vibration data of the bearing with outer race fault of 1.3 mm and the motor operated under the load of 100watt.
25. 1.3outer-200 watt: Triaxial vibration data of the bearing with outer race fault of 1.3 mm and the motor operated under the load of 200watt.
26. 1.3outer-300 watt: Triaxial vibration data of the bearing with outer race fault of 1.3 mm and the motor operated under the load of 300watt.
27. 1.5inner-100 watt: Triaxial vibration data of the bearing with inner race fault of 1.5 mm and the motor operated under the load of 100watt.
28. 1.5inner-200 watt: Triaxial vibration data of the bearing with inner race fault of 1.5 mm and the motor operated under the load of 200watt.
29. 1.5inner-300 watt: Triaxial vibration data of the bearing with inner race fault of 1.5 mm and the motor operated under the load of 300watt.
30. 1.5outer-100 watt: Triaxial vibration data of the bearing with outer race fault of 1.5 mm and the motor operated under the load of 100watt.
31. 1.5outer-200 watt: Triaxial vibration data of the bearing with outer race fault of 1.5 mm and the motor operated under the load of 200watt.
32. 1.5outer-300 watt: Triaxial vibration data of the bearing with outer race fault of 1.5 mm and the motor operated under the load of 300watt.
33. 1.7inner-100 watt: Triaxial vibration data of the bearing with inner race fault of 1.7 mm and the motor operated under the load of 100watt.
34. 1.7inner-200 watt: Triaxial vibration data of the bearing with inner race fault of 1.7 mm and the motor operated under the load of 200watt.
35. 1.7inner-300 watt: Triaxial vibration data of the bearing with inner race fault of 1.7 mm and the motor operated under the load of 300watt.
36. 1.7outer-100 watt: Triaxial vibration data of the bearing with outer race fault of 1.7 mm and the motor operated under the load of 100watt.
37. 1.7outer-200 watt: Triaxial vibration data of the bearing with outer race fault of 1.7 mm and the motor operated under the load of 200watt.
38. 1.7outer-300 watt: Triaxial vibration data of the bearing with outer race fault of 1.7 mm and the motor operated under the load of 300watt.

The dataset includes a total of 38 files of the bearing conditions including healthy bearing and bearings with faults of different severity levels which were operated under three different load conditions.

## 2 Experimental Design, Materials and Methods

### 2.1 Experimental system

**Fig. 1:**
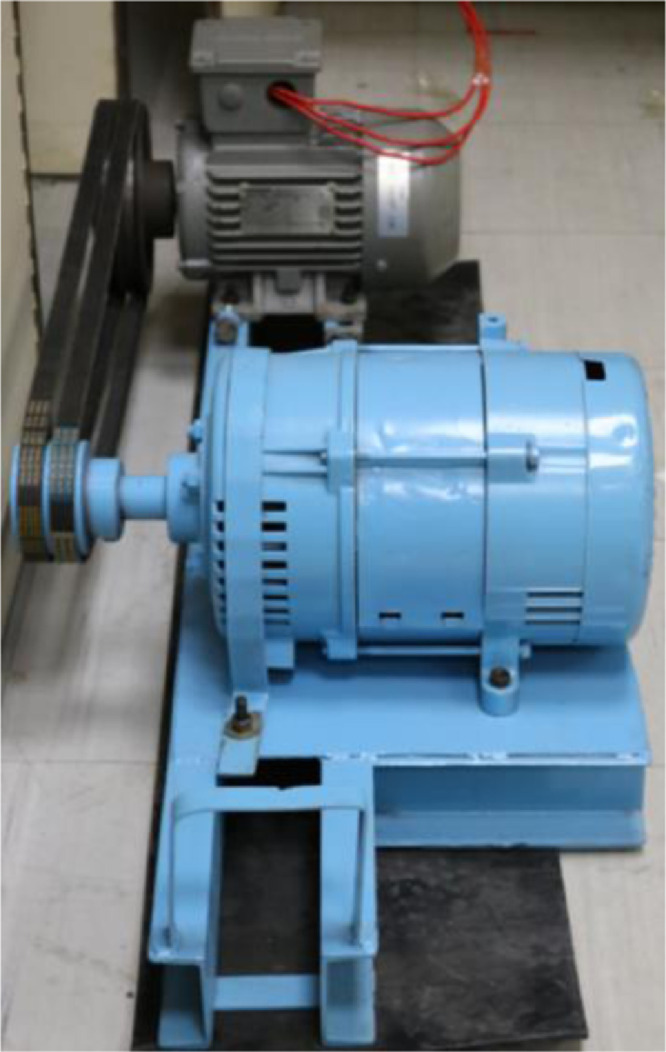
Experimental setup for bearing fault diagnosis including a three phase motor and an alternator coupled through a belt.

To carry out this research, a test rig comprising of a three phase induction motor and an alternator with variable electrical load system was utilized as shown in Fig 1. The motor and the alternator were coupled via a belt. The motor includes two ball bearings on the drive end and the non-drive end and the structural parameters of the bearing are given in Table 1. Initially, the vibration data was collected for the healthy bearing. Then, the bearing of the drive end was replaced multiple times with the faulty bearings having different severity levels of faults including inner race and outer race faults.

**Table 1:** Bearing Parameters.

Bearing Model	Outer Diameter	Inner Diameter	Width	No. of Balls
6204-2Z/C3	47 mm	20 mm	14 mm	8

A MEMS based accelerometer (ADXL355) was attached to the housing of the three phase induction motor near to the drive-end bearing. The accelerometer was attached to the housing of the motor in such a way that the z-axis was directed upwards. The system was programmed using the LabVIEW software. The drive-end bearing was replaced each time after establishing a time-series dataset of each bearing condition.

### 2.2 Data acquisition system and settings

The data was acquired using a data acquisition system comprising of National Instruments (NI) myRIO board and a MEMs based triaxial accelerometer. The NI myRIO board facilitated high speed data acquisition owing to the FPGA capabilities incorporated in it. The triaxial vibration data was acquired at a sampling rate of 10 kHz at the rate of 1000 samples per channel.

### 2.3 Datasets

The dataset is stored on the Mendeley data platform with the title of “Triaxial Bearing Vibration Dataset of Induction Motor under Varying Load Conditions”. A total of 38 datasets have been established and are available at:http://dx.doi.org/10.17632/fm6xzxnf36.2. Each dataset is acquired under three load conditions which include (i) 100W, (ii) 200W, and (iii) 300W loads. The fault severity levels include 0.7mm, 0.9mm, 1.1mm, 1.3mm, 1.5mm, and 1.7mm. The bearing conditions include healthy bearing and bearings with inner race faults and outer race faults of different severity levels.

## CRediT Author Statement

**Dileep Kumar:** Conceptualization, Methodology, Data Acquisition, and Formal Analysis; **Sanaullah Mehran:** Methodology, Data curation and Writing-Original draft; **Muhammad Zakir Hussain:** Methodology, Writing and Investigation; **Majid Hussain:** Software, Methodology, and Data acquisition; **Bhawani Shankar Chowdhry:** Project administration, Supervision and Validation; **Tanweer Hussain:** Supervision, Reviewing and Editing.

## Ethics Statement

The NCRA Condition Monitoring Systems Lab, Mehran University of Engineering and Technology Jamshoro, Pakistan has given the consent that the datasets may be publicly-released as part of this publication.

## Declaration of Competing Interest

The authors declare that they have no known competing financial interests or personal relationships that could have appeared to influence the work reported in this paper.

## Data Availability

Triaxial Bearing Vibration Dataset of Induction Motor under Varying Load Conditions (Original data) (Mendeley Data).
